# Cadence Control Training Alters Patellofemoral Joint Mechanics and Lower‐Limb Joint Work Distribution in Runners

**DOI:** 10.1155/abb/5515480

**Published:** 2026-08-31

**Authors:** Gang Ma, Chuanbao Cao, Xu Wang, Hai Bo, Peng Peng, Kaili Shao

**Affiliations:** ^1^ Logistics University of People’s Armed Police Force, Tianjin, China; ^2^ Hebei Sport University, Shijiazhuang, China

**Keywords:** cadence control training, joint work contribution, patellofemoral joint stress

## Abstract

**Background:**

This study aimed to examine the effects of a 12‐week cadence control training program on patellofemoral joint mechanics and lower‐limb joint work distribution in runners, thereby providing a theoretical basis for optimizing running strategies and reducing running‐related injury (RRI) risk.

**Methods:**

A total of 44 recreational runners were randomly assigned to a control (*n* = 22) and an experimental group (EG; *n* = 22). The former performed conventional running training, while the latter underwent cadence control training for 12 weeks. Three‐dimensional (3D) kinematic and kinetic data were collected before and after the intervention using the Simi Motion capture system and Kistler force platforms, respectively. Patellofemoral joint mechanical parameters and joint work contribution ratios were subsequently calculated.

**Results:**

Posttraining, the EG exhibited a significant increase in cadence and positive ankle joint work contribution and a significant reduction in patellofemoral joint stress (PFJS), compared with pretraining values.

**Conclusions:**

Twelve weeks of cadence control training significantly modified the patellofemoral joint mechanics and joint work distribution in runners. An increased cadence reduced patellofemoral joint loading and induced a distal shift in the relative contribution of lower‐limb joint work from proximal to distal segments, which may represent a biomechanical mechanism underlying the alleviation of knee joint load. Therefore, cadence‐increasing running strategies are recommended, particularly for runners experiencing patellofemoral pain (PFP), to mitigate the risk of knee joint injury.

## 1. Introduction

Running represents one of the most common forms of physical activity. However, given its repetitive nature and weight‐bearing demands, running‐related injuries (RRIs) have a higher incidence than in most other sports [1]. Epidemiological studies have reported that more than 50% of runners experience RRIs [2].

Running injuries are strongly associated with repetitive high‐impact forces acting on the lower limbs [3]. Therefore, mitigating lower‐limb impact forces during running is crucial for injury prevention. The patella plays an essential biomechanical role by increasing the quadriceps’ moment arm and improving the efficiency of knee extension [4]. However, abnormal patellar tracking, muscular imbalance between the vastus medialis and vastus lateralis, and overtraining can increase patellofemoral joint stress (PFJS), which leads to patellofemoral pain (PFP) [5]. Reportedly, PFP is one of the most prevalent types of knee injury among runners, affecting up to 25% of runners [6].

Cadence manipulation refers to a simple yet effective strategy for altering lower‐limb loading during running [7]. It requires no specialized equipment, can be easily implemented with a simple metronome, and is readily adopted by runners. At a constant running speed, increasing step cadence by 5%–10% can effectively reduce the vertical ground reaction force (vGRF) [8] and knee flexion angle and knee joint load [9]. Specifically, a 10% reduction in step length decreased PFJS per step by 22.2%, while a 10% increase in step length increased PFJS per step by 31%, directly demonstrating that step length manipulation effectively alters patellofemoral joint loading [9]. Such adjustments in running strategy may therefore help reduce the risk of lower‐limb joint injuries. However, running involves coordinated motion across the hip, knee, and ankle joints. Analysis of joint work distribution provides insights into intersegmental coordination and compensatory mechanisms within the kinetic chain [10]. Consequently, cadence modification may alter the distribution of lower‐limb joint work, thereby reducing PFJS during running.

Although previous studies have demonstrated the effects of cadence manipulation on joint loading, most were limited to acute biomechanical responses [8, 9]. Consequently, the long‐term training effects of cadence control on patellofemoral joint mechanics and lower‐limb joint work distribution remain unclear. In particular, no randomized controlled trial has examined the chronic adaptation of joint work redistribution following a multiweek cadence control training program in recreational runners.

Against this backdrop, our study investigated the effects of a 12‐week cadence control training program on patellofemoral joint mechanics and joint work distribution in runners, providing a biomechanical basis for optimizing running strategies and reducing the risk of RRIs. We hypothesized that, compared with conventional running training, the cadence control training would (1) significantly reduce PFJS and (2) alter the lower‐limb joint work distribution.

## 2. Materials and Methods

### 2.1. Participants

This study adhered to the Declaration of Helsinki and was approved by the Ethics Committee of Hebei Sport University (No. 2024026). An a priori power analysis was performed using 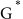
Power software (Version 3.1.9.2) [11], with a significance level (*α*) of 0.05, statistical power (1–*β*) of 0.8, and an effect size of 0.5. The analysis yielded a minimum required sample size of 34 participants. To ensure adequate statistical power and account for potential attrition, we initially recruited 48 male recreational runners from a local university and randomly assigned them to either a control group (CG, *n* = 24) or an experimental group (EG, *n* = 24) via a lottery‐based randomization procedure. Eligible participants were between 20 and 30 years of age, ran at least 20 km per week, and were able to complete a 12‐week training program. All participants habitually used a rearfoot strike pattern and were accustomed to outdoor running, with no regular treadmill training experience. They had at least 1 year of running experience. Exclusion criteria included any history of a lower‐limb musculoskeletal injury or a gait‐affecting condition within the past 6 months. The CG performed 12 weeks of conventional running training, while the EG underwent 12 weeks of cadence control training. During the intervention, one participant withdrew due to health reasons and three were excluded for incomplete training compliance, resulting in a final sample of 44 participants (Table 1). All participants were informed of the experimental procedures and potential risks and were requested to provide written informed consent before participation.

**Table 1:** Participant characteristics.

Group	Age	Height	Weight	BMI	Running volume
CG(*n* = 22)	23.682 ± 2.124	171.164 ± 5.523	69.267 ± 7.691	23.591 ± 1.795	27.455 ± 2.283
EG(*n* = 22)	24.636 ± 2.013	169.604 ± 4.876	69.495 ± 6.434	24.149 ± 1.894	26.500 ± 2.686
*t*	−1.530	0.993	−0.106	−1.004	1.270
*p*	0.134	0.327	0.916	0.321	0.211

### 2.2. Protocol

#### 2.2.1. Testing Procedure

The participants wore standardized athletic attire throughout all testing sessions. Anthropometric data (including height, weight, and dominant leg) were initially recorded, followed by a 5 min warm‐up session. The dominant limb was determined by kicking a soccer ball [10]. After the warm‐up, 41 active infrared‐emitting markers were affixed to anatomical landmarks of the head, trunk, pelvis, bilateral upper limbs, bilateral lower limbs, and feet in accordance with established protocols [12]. Then, the participants ran along a runway at a controlled speed of 12 km/h (±5%) to reduce the influence of running speed on the experimental results [13]. Kinematic data were captured using a three‐dimensional (3D) motion analysis system (Simi Motion System, Germany; sampling rate: 100 Hz), and GRFs were simultaneously collected using a Kistler 3D force platform (Kistler‐9287BA, Switzerland; sampling rate: 1000 Hz). All kinematic and kinetic data were synchronized for subsequent biomechanical analysis. Three valid trials were collected for each participant.

#### 2.2.2. Training Intervention

Before the intervention, each participant’s preferred cadence was determined. A wireless motion sensor (BTS G‐WALK, Italy) was positioned at the level of the sacrum, and at their self‐selected comfortable speed and cadence, the participants completed three trials of treadmill running. The average cadence from the three trials was defined as the preferred cadence. Both groups completed a 12‐week running program consisting of three sessions per week, with each lasting 45 min, to ensure equivalent training volume between groups [14]. The CG maintained their preferred cadence throughout the training period, and the EG performed cadence control training using a metronome, increasing their running cadence by 7.5% relative to the baseline [15]. Each session was supervised by professional coaches, and participants were instructed not to engage in any additional running training outside the 3 weekly sessions.

### 2.3. Data Processing

Raw kinematic and kinetic data were processed using Simi Motion and imported into Visual 3D for filtering, normalization, and time–percentage standardization. Marker trajectory and GRF data were low‐pass filtered using a fourth‐order Butterworth digital filter with cutoff frequencies of 6 Hz and 50 Hz, respectively [16]. Normalization of kinetic data to body weight was determined for intersubject comparison. The stance phase of running (defined as the period from initial foot contact to the toe‐off of the dominant leg) was selected for analysis.

### 2.4. Parameters

#### 2.4.1. Patellofemoral Joint Mechanics

Patellofemoral joint mechanics were computed based on a previously established biomechanical model [12].

1. Quadriceps force (QF) [12]:

QF was defined as the ratio of the knee extension moment to the effective moment arm of the quadriceps (LA), as shown in Equation (1), where *M*_EXT_ represents the knee extension moment, and LA denotes the effective moment arm of the quadriceps. LA was modeled as a piecewise linear function of the knee angle (Equation (2)):

(1) QFθi=MEXTθi/ LAθi×0.01,

(2) LA0.0.363.0θ+030≤θ<−0.0435.4θ+3060≤θ<−0.0274.3θ+6090≤θ<2.090≤θ.

2. Patellofemoral joint force (PFJF) [12]:

PFJF was calculated as a function of the QF and angle *β*, as shown in Equation (3), where *β* (°) refers to the angle between the QF line and the patellar tendon line of action (Equation (4)):

(3) PFJF=2QFsinβ/2,

(4) β=30.460.53+θ.

3. PFJS [12]:

The PFJS was defined as the ratio of PFJF to the patellofemoral contact area (PFCA), as shown in Equation (5), where PFCA represents the contact area between the patella and femur (Equation (6)):

(5) PFJSθi=PFJFθi/PFCAθi,

(6) PFCAθi=0.07810.6763151.75×θi2+×θi+.

#### 2.4.2. Joint Work Distribution

1. Joint work [16]:

Joint power (*P*_*i*_) was defined as the product of the joint moment (*M*_*i*_) and joint angular velocity (*ω*_*i*_) (Equation (7)); joint work was obtained through the integration of joint power over time during the stance phase, as shown in Equations (8) and (9). The net joint work (*W*_net_) was calculated as the algebraic sum of the positive (*W*_*+*_) and negative work (*W*_-_) components (Equation (10)):

(7) Pi=Mi×ωi,

(8) W+=∫t1t2Pdt    p>0,

(9) W−=∫t1t2Pdt    p>0,

(10) Wnet=W++W−.

2. Joint work contribution (Con_joint_) [16]:

The contribution of each lower‐limb joint to total net joint work (*Con*_*joint*_) was expressed as the ratio of the net work of an individual joint to the sum of the net work of all three major lower‐limb joints (hip, knee, and ankle) (Equation (11)):

(11) Conjoint=Wjoint netWhip net+Wknee net+Wankle net×100%.

### 2.5. Statistical Analysis

All statistical analyses were performed using SPSS 26.0. The Shapiro–Wilk test was conducted to assess data normality, and Levene’s test was used to assess the homogeneity of variances. The results confirmed that all data met the normality and homogeneity assumptions. Two‐way mixed‐design ANOVA was employed to examine the effects of training (pre‐ vs. postintervention) and group (CG vs. EG) on patellofemoral joint mechanics and joint work distribution. When a significant interaction was found, simple effect analysis was performed. All post hoc pairwise comparisons were adjusted using Bonferroni correction. The statistical significance level was set at *p*  < 0.05.

## 3. Results

### 3.1. Effects of Cadence Control Training on Running Gait

Two‐way mixed‐design ANOVA revealed a significant interaction effect for cadence (*p* = 0.000, *η^2^*_*p*_ = 0.473). Simple effects analysis showed that, compared with preintervention, the EG had a significant increase in cadence after the intervention (146.14 ± 6.95 vs. 149.08 ± 8.00, *p* = 0.001, 95% CI = −4.585 to 1.295) (Table 2).

**Table 2:** Changes in running gait parameters before and after training.

Gait parameters	CG		EG	
	Preintervention	Postintervention	Preintervention	Postintervention
Step length (m)	1.692 ± 0.288	1.642 ± 0.308	1.703 ± 0.333	1.505 ± 0.265
Step width (m)	0.322 ± 0.043	0.315 ± 0.047	0.303 ± 0.051	0.313 ± 0.055
Cadence (step/min)	148.225 ± 7.525	147.785 ± 6.904	146.141 ± 6.945	149.081 ± 7.998a

*Note:* “a” indicates a significant difference between pre‐ and postintervention within the same group.

### 3.2. Effects of Cadence Control Training on Patellofemoral Joint Mechanics

**Figure 1:**
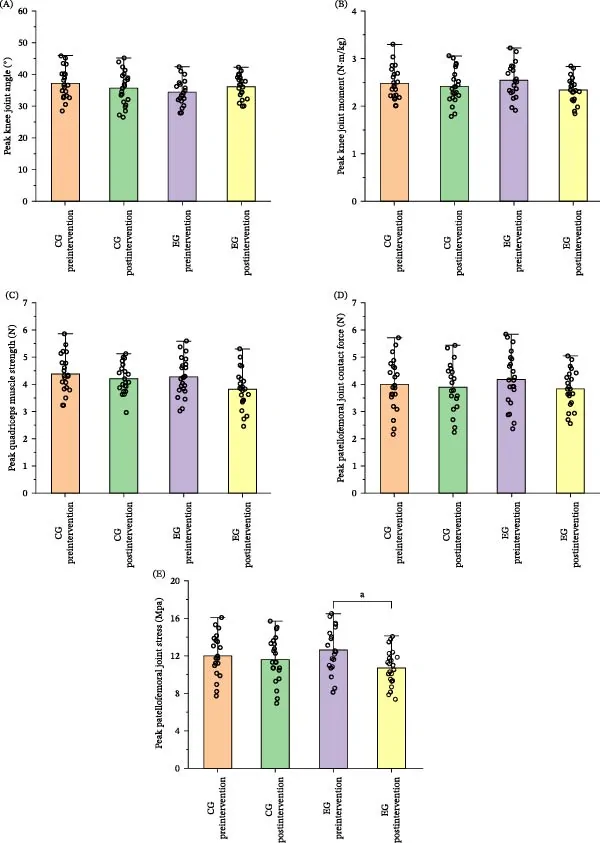
Modifications in patellofemoral joint mechanics before and after training. (A) Peak knee flexion angle, (B) peak knee extension moment; (C) peak QF; (D) peak PFJF; (E) peak PFJS. “a” indicates a significant difference between pre‐ and postintervention within the same group.

Two‐way mixed‐design ANOVA revealed a significant interaction effect for PFJS (*p* = 0.027, *η^2^*_*p*_ = 0.211). Simple effects analysis showed that, compared with preintervention, *PFJS* in the EG significantly decreased after the intervention (12.63 ± 2.31 vs. 10.71 ± 1.88, *p* = 0.002, 95% CI = 0.796–3.040) (Figure 1).

### 3.3. Effects of Cadence Control Training on Lower‐Limb Joint Work Distribution

**Figure 2:**
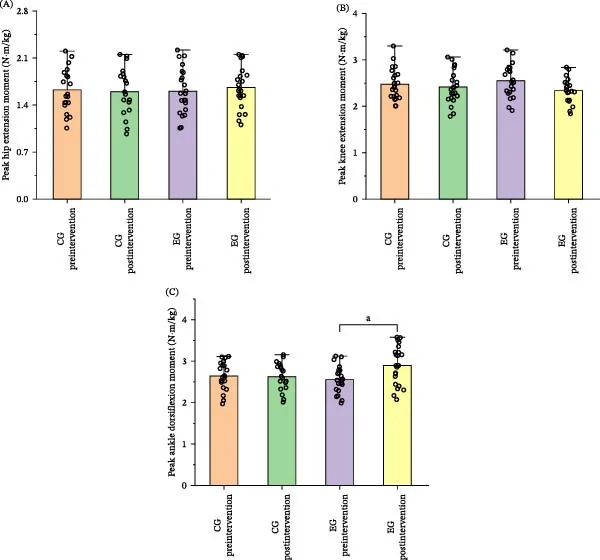
Changes in joint moments before and after training. (A) Peak hip extension moment; (B) peak knee extension moment; (C) peak ankle dorsiflexion moment. “a” indicates a significant difference between pre‐ and postintervention within the same group.

Two‐way mixed‐design ANOVA revealed a significant interaction effect for peak ankle dorsiflexion moment (*p* = 0.017, *η^2^*_*p*_ = 0.242). Simple effects analysis showed that, compared with preintervention, peak ankle dorsiflexion moment in the EG significantly increased after the intervention (2.56 ± 0.32 vs. 2.90 ± 0.47, *p* = 0.020, 95% CI = −0.623 to −0.059) (Figure 2).

**Figure 3:**
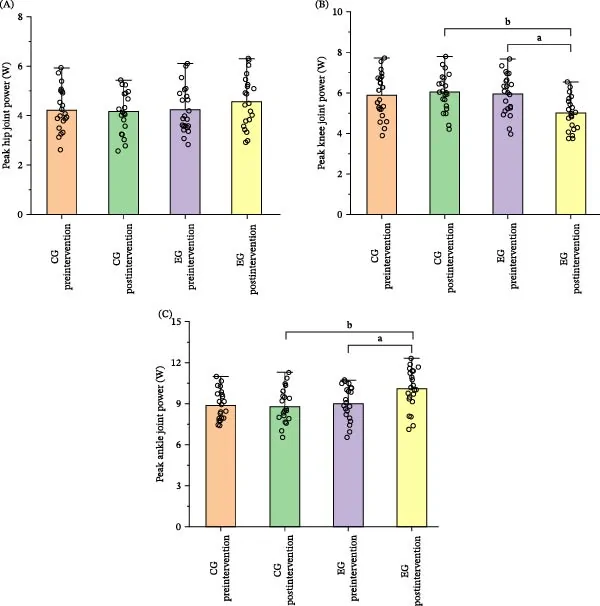
Alterations in lower‐limb joint power before and after training. (A) Peak hip extension power; (B) peak knee extension power; (C) peak ankle dorsiflexion power. “a” indicates a significant difference between pre‐ and postintervention within the same group; “b” denotes a significant difference between groups at the same time point.

Two‐way mixed‐design ANOVA revealed significant interaction effects for peak knee and ankle joint power (*p* = 0.016, *η^2^*_*p*_ = 0.248; *p* = 0.024, *η^2^*_*p*_ = 0.220). Compared with preintervention values, the EG showed a significant increase in peak ankle joint power (9.01 ± 1.27 vs. 10.09 ± 1.47, *p* = 0.012, 95% CI = −1.890 to −0.265) and a significant decrease in peak knee joint power (5.94 ± 1.00 vs. 5.01 ± 0.82, *p* = 0.003, 95% CI = 0.367–1.509) following 12‐week cadence control training. Compared with the CG postintervention, the EG showed significantly higher ankle joint power (8.81 ± 1.25 vs. 10.09 ± 1.47, *p* = 0.007, 95% CI = −2.179 to −0.381) and significantly lower peak knee joint power (6.03 ± 0.93 vs. 5.01 ± 0.82, *p* = 0.000, 95% CI = 0.529–1.516) (Figure 3).

Two‐way mixed‐design ANOVA revealed significant interaction effects for ankle positive work and total work (*p* = 0.011, *η^2^*_*p*_ = 0.270; *p* = 0.006, *η^2^*_*p*_ = 0.309). Following intervention, the EG showed significant increases in ankle joint positive work (0.60 ± 0.08 vs. 0.70 ± 0.10, *p* = 0.002, 95% CI = −0.160 to −0.043) and net work (0.03 ± 0.13 vs. 0.16 ± 0.07, *p* = 0.002, 95% CI = −0.024 to 0.089) compared with pretraining values. In addition, postintervention comparisons between groups revealed significantly higher ankle joint positive work (0.62 ± 0.09 vs. 0.70 ± 0.10, *p* = 0.008, 95% CI = −0.143 to −0.024) and net work (0.03 ± 0.13 vs. 0.16 ± 0.07, *p* = 0.000, 95% CI = −0.202 to −0.066) in the EG than in the CG (Table 3).

**Table 3:** Changes in lower‐limb joint work before and after training.

Joint work	CG		EG	
	Preintervention	Postintervention	Preintervention	Postintervention
Hip joint positive work (J)	0.225 ± 0.065	0.225 ± 0.065	0.219 ± 0.009	0.244 ± 0.064
Hip joint negative work (J)	−0.071 ± 0.019	−0.069 ± 0.025	−0.064 ± 0.015	−0.068 ± 0.018
Hip joint net work (J)	0.154 ± 0.089	0.157 ± 0.077	0.155 ± 0.083	0.176 ± 0.070
Knee joint positive work (J)	0.375 ± 0.040	0.349 ± 0.045	0.375 ± 0.046	0.326 ± 0.047
Knee joint negative work (J)	−0.529 ± 0.077	−0.505 ± 0.074	−0.526 ± 0.072	−0.461 ± 0.063
Knee joint net work (J)	−0.153 ± 0.080	−0.156 ± 0.069	−0.151 ± 0.062	−0.135 ± 0.063
Ankle joint positive work (J)	0.630 ± 0.089	0.616 ± 0.090	0.598 ± 0.080	0.699 ± 0.102 ab
Ankle joint negative work (J)	−0.575 ± 0.098	−0.588 ± 0.088	−0.565 ± 0.109	−0.537 ± 0.093
Ankle joint net work (J)	0.055 ± 0.137	0.028 ± 0.126	0.032 ± 0.127	0.162 ± 0.072 ab

*Note:* “a” indicates a significant difference between pre‐ and postintervention within the same group; “b” denotes a significant difference between groups at the same time point.

**Figure 4:**
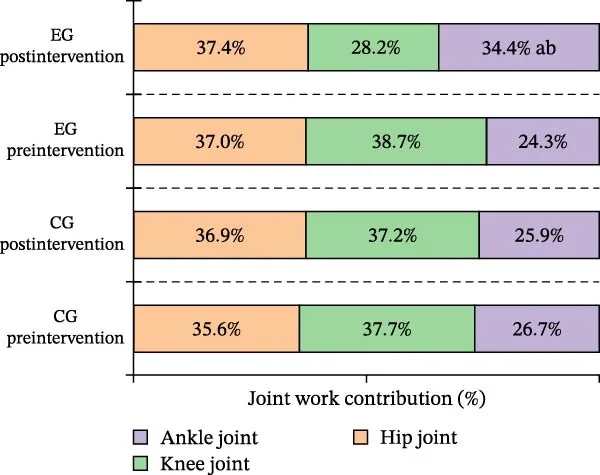
Changes in lower‐limb joint work contribution before and after training. “a” indicates a significant difference between pre‐ and postintervention within the same group; “b” denotes a significant difference between groups at the same time point.

Two‐way mixed‐design ANOVA revealed a significant interaction effect for ankle joint work contribution (*p* = 0.031, *η^2^*_*p*_ = 0.202). After the 12‐week training, the EG demonstrated a significant increase in ankle joint work contribution (0.24 ± 0.13 vs. 0.34 ± 0.11, *p* = 0.021, 95% CI = −0.185 to −0.017) relative to baseline. Moreover, compared with the CG postintervention, the EG showed significantly higher ankle joint work contribution (0.26 ± 0.13 vs. 0.34 ± 0.11, *p* = 0.016, 95% CI = −0.153 to −0.018) (Figure 4).

## 4. Discussion

In this study, the effects of a 12‐week cadence control training program on patellofemoral joint mechanics and lower‐limb joint work distribution in runners were investigated. Our findings include the following: After the intervention, cadence significantly increased, patellofemoral joint loading was significantly reduced, and the relative contributions of lower‐limb joints to total mechanical work were altered.

After 12 weeks of cadence control training, participants in the EG demonstrated a significant increase in cadence. Increasing the cadence by ~5%–10% can effectively reduce GRFs and joint loading during running [8]. Following the cadence adjustment protocol proposed by Willy et al. [15], the present study adopted a 7.5% increase in preferred cadence as the intervention target during training, with metronome guidance at self‐selected speeds. The 2% increase observed after intervention reflects the spontaneous transfer to a fixed‐speed condition without auditory feedback. The observed postintervention increase in cadence is consistent with prior findings [17], suggesting that cadence control training can lead to an increase in cadence even in the absence of external feedback.

Patellofemoral joint loading showed a significant reduction following 12 weeks of cadence control training. PFP represents one of the most common types of RRIs [5]. Excessive PFJS is a primary contributing factor to PFP as increased compressive stress on the joint can lead to cartilage degeneration and consequently elevate the risk of developing PFP syndrome (PFPS) [12]. Therefore, reduction of PFJS during running is critical for the prevention and management of PFPS. In this work, significantly lower PFJS was observed after 12 weeks of cadence control training compared with pretraining values. PFJS is determined by the PFJF and PFCA. In this study, the observed reduction in QF contributed to the decrease in PFJF; the lack of significant change in PFCA may be related to the individual knee anatomy. Therefore, the reduction in joint stress was primarily attributable to the decrease in the PFJF. Runners with PFPS often adopt a self‐optimized gait strategy characterized by reduced QF, achieved by lowering the knee extension moment [18].

Following the 12‐week cadence control training, notable changes were observed in the distribution of lower‐limb joint work. Modification of the running technique effectively redistributes or reduces joint loading [19]. Among various gait retraining strategies, cadence adjustment is recognized as one of the simplest and most effective methods for optimizing running performance and minimizing injury risk. Evidence suggests a strong association between the cadence and the incidence of RRIs [20]. Runners with low cadence tend to experience a higher peak vGRF, which in turn increases the risk of RRIs [20]. During running, the lower‐limb joints work synergistically to absorb, store, and generate mechanical energy [21]. In the present work, cadence control training resulted in significant reductions in knee joint power, accompanied by significant increases in ankle‐joint moment, power, and positive and net work. These findings suggest that with increased cadence, the knee joint may reduce its energy absorption to minimize loading and injury risk, and the ankle joint compensates by increasing positive work output to maintain running velocity.

Furthermore, coordination among lower‐limb joints contributes to the understanding of injury mechanisms. Our study results demonstrate that increased cadence led to a redistribution of mechanical work among the lower‐limb joints. Specifically, after 12 weeks of cadence control training, the contribution of the ankle joint significantly increased, which indicates a distal shift in joint work contribution from the proximal to distal segments. In OpenSim simulations, the stronger limb exhibited greater peak knee joint moments and higher muscle activation at the ankle, suggesting the existence of a load‐redistribution mechanism between the knee and ankle joints [22]. This shift reflects a potential redistribution of the mechanical load within the kinetic chain. The distal redistribution of mechanical work may reduce energy absorption at the knee, thereby potentially decreasing knee joint loading. Whether such biomechanical adjustments are accompanied by changes in motor control or neuromuscular coordination requires further investigation using electromyography.

## 5. Limitations

This study encountered several limitations. First, we did not perform intersession reliability testing, and the intervention was limited to a single cadence adjustment strategy in healthy runners only without including a PFP group. Second, only data on the dominant leg were analyzed, which may fail to fully account for potential asymmetries in bilateral lower‐limb motor control during running. Third, we did not measure subject‐specific patellofemoral anatomy, and the observed distal shift of load toward the ankle could increase stress on other structures. Future research should include symptomatic populations, bilateral data, anatomical assessments, and evaluation of potential trade‐offs to better understand cadence modification.

## 6. Conclusion

Twelve weeks of cadence control training altered patellofemoral joint mechanics and joint work distribution in runners. An increase in running cadence effectively reduced patellofemoral joint loading and induced a distal redistribution of lower‐limb joint work contribution from the knee to the ankle, which may represent a biomechanical mechanism for alleviating knee joint stress. Therefore, cadence‐increasing running strategies are recommended, particularly for runners with PFP, as an effective approach to reduce knee joint load and mitigate the risk of RRIs.

## Funding

This research was funded by the Key Research and Development Plan of Hebei Province (Grant 22375701D), the National Defense Science and Technology Innovation Project (Grant ZZKY20253137), and the Scientific Research Team Project of Logistics University of People’s Armed Police Force.

## Conflicts of Interest

The authors declare no conflicts of interest.

## Data Availability

The data that support the findings of this study are available from the corresponding author upon reasonable request.
